# Cellular immune responses of bovine polymorphonuclear neutrophils to *Calicophoron daubneyi*

**DOI:** 10.3389/fimmu.2025.1515419

**Published:** 2025-02-13

**Authors:** Liliana M. R. Silva, Sara López-Osorio, Raquel Peixoto, Ershun Zhou, Gabriel Espinosa, Ulrich Gärtner, Anja Taubert, Iván Conejeros, Carlos Hermosilla

**Affiliations:** ^1^ Institute of Parasitology, Biomedical Research Center Seltersberg, Justus Liebig University Giessen, Giessen, Germany; ^2^ Egas Moniz Center for Interdisciplinary Research (CiiEM), Egas Moniz School of Health and Science, Almada, Portugal; ^3^ Mediterranean Institute for Agriculture, Environment and Development (MED) and Global Change and Sustainability Institute (CHANGE), University of Évora, Évora, Portugal; ^4^ CIBAV Research Group, Faculty of Agrarian Sciences, University of Antioquia, Medellín, Colombia; ^5^ College of Life Sciences and Engineering, Foshan University, Foshan, Guangdong, China; ^6^ Institute of Anatomy and Cell Biology, Faculty of Human Medicine, Justus Liebig University Giessen, Giessen, Germany

**Keywords:** fluke, *Calicophoron daubneyi*, NETosis, extracellular trap formation, degranulation, bovine neutrophils

## Abstract

*Calicophoron daubneyi* infections have increased in Europe, being more frequent than fasciolosis in some areas. Infection occurs once definitive hosts ingest encysted metacercariae present on vegetation. Following excystation, juvenile flukes penetrate the small intestinal mucosa and migrate into the rumen where adults mature. Throughout the somatic migration, juveniles come across different microenvironments and tissues and encounter host leukocytes. Besides phagocytosis, production of reactive oxygen species (ROS) and degranulation, polymorphonuclear neutrophils also cast neutrophil extracellular traps (NETs), which can entrap several parasite species, including the closely related liver fluke *Fasciola hepatica*. In this study, we analyzed whether *in vitro* exposure of bovine neutrophils to *C. daubneyi* antigen (*Cd*Ag) and eggs triggered neutrophils activation and NET formation. Results on scanning electron microscopy (SEM) and immunofluorescence analyses show weak formation of short spread NETs upon *Cd*Ag stimulation, corroborated by increased extracellular DNA measurements. Likewise, early NETosis was confirmed via nuclear area expansion assays. Bovine neutrophil stimulation with *Cd*Ag 100 µg/mL concentration led to a significant increase in oxygen consumption rates (*p* = 0.0152) and extracellular acidification rates (*p* = 0.0022), while lower concentrations of *Cd*Ag (10 µg/mL) failed to induce neutrophil activation, suggesting a dose dependent response. Both intra- and extracellular ROS production was not affected by any *Cd*Ag concentration here studied. Bovine neutrophil total adenosine triphosphate concentration significantly decreased after exposure to *Cd*Ag 100 µg/mL, in line to the observed with the positive control (phorbol myristate acetate/ionomycin). In summary, *C. daubneyi* activates bovine neutrophils with rather weak responses, which might suggest that the release of *C. daubneyi*-specific molecules (i.e. excretory-secretory antigens, proteases, or nucleases) could interfere with neutrophil-related effector mechanisms. Further *ex vivo* analyses will clarify if such mechanisms are also involved in pathogenesis of paramphistomosis by demonstrating neutrophil recruitment into affected intestinal mucosa.

## 1 Introduction

*Calicophoron daubneyi* are pink pear-shaped trematodes present in the rumen and reticulum of domestic and wild ruminants such as cattle, buffalo, sheep, goat, deer, and bison, causing paramphistomosis worldwide (1–8), especially in tropical and subtropical climates. Nonetheless, *C. daubneyi* infections considerably increased in the last years in Western Europe, in both sheep and cattle, being even more common than *Fasciola hepatica* in many geographic areas (8). Several reports from Western Europe confirmed via molecular tools *C. daubneyi* as the primary trematode species present in ruminants (9). Such rise in European countries might be related to the characteristic warm wet summers and mild winters of many areas that are optimal for its gastropod intermediate host, *Galba truncatula* (9). Clinical paramphistomosis is mostly caused by immature stages during the migratory phase from the small intestine lumen to the submucosa (10), leading to lethargy, submandibular edema, and tissue damage (8, 11). In heavy infections, hemorrhagic enteritis, necrosis, diarrhea, dehydration, cachexia, and even death may occur (12, 13). In contrast, the ruminal phase of infection is less pathogenic, but immature stages ultimately induce rumen papillae atrophy and ruminitis (14).

**Figure 1:**
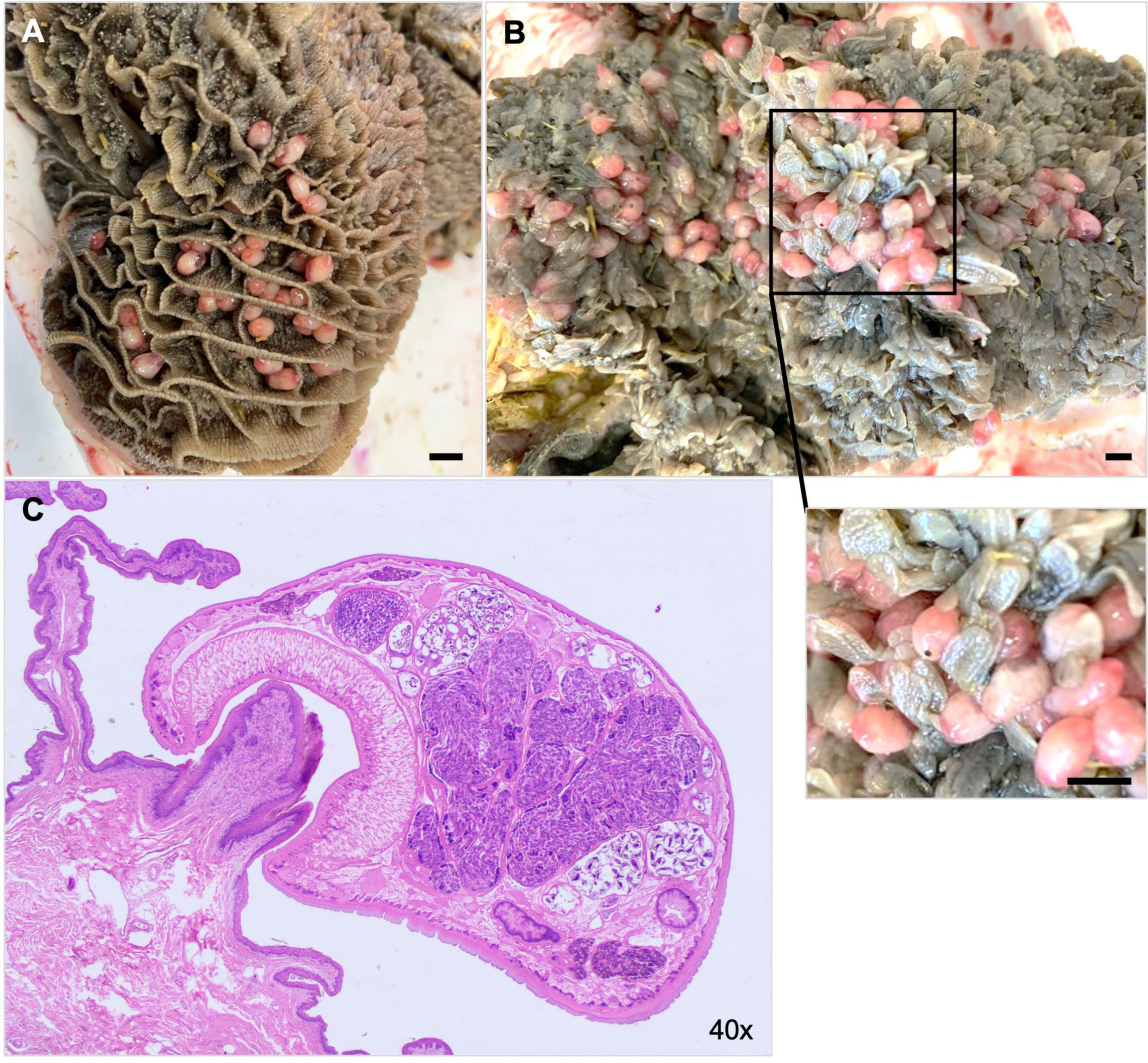
*Calicophoron daubneyi* adults parasitize the rumen of cattle and other ruminants. **(A)** *C*. *daubneyi* mature adults in the rumen of cattle. **(B)** Pear-shaped flukes attached to rumen papilla. **(C)** Adult fluke attached firmly to a rumen papilla via muscular posterior acetabulum.

As already stated*, C. daubneyi* has an indirect life cycle with freshwater snails (families Lymnaeidae and Planorbidae, typically *G. truncatula*) acting as obligate intermediate hosts (3, 8, 15). Definitive hosts are infected after ingestion of encysted metacercariae present on vegetation. After excystation in the duodenum, newly excysted juveniles (NEJ) feed on mucosa until they are prepared to penetrate the small intestinal mucosa and retrogradely migrate in the duodenal- and abomasal mucosa to the rumen where mature adults attach firmly to the rumen wall or papillae (**Figures 1A, B**) by the posterior muscular acetabulum (**Figure 1C**) (8, 12). During migration, *C. daubneyi* NEJ may encounter leukocyte populations of host innate immune system, including polymorphonuclear neutrophils (PMN) which are activated when pathogen recognition receptors (PRRs) bind to parasite-specific molecules (16).

Neutrophils are the most abundant leukocytes in blood and lymph and first ones being attracted to sites of infection. Neutrophils may target pathogens by phagocytosis, secretion of pro-inflammatory cytokines and chemokines, production of reactive oxygen species (ROS), degranulation of antimicrobial peptides, and formation of neutrophil extracellular traps (NETs) (16, 17), playing a decisive role in the development of host innate and adaptative immune responses (18).

NETs consist of nuclear and mitochondrial DNA decorated with granule proteins [e. g. citrunillated histones (H1, H2A/H2B, H3, H4), neutrophil elastase (NE), myeloperoxidase (MPO), lactoferrin, gelatinase, pentraxin, cathepsin G (Cat G), cathelicidin (LL37) among others] with recognized antimicrobial properties (19, 20). Over the last years, distinct phenotypes of NETs have been described in response to several pathogen and molecular stimuli. The main phenotypes include: *i*) diffuse NETs (*diff*NETs), *ii*) spread NETs (*spr*NETs), and *iii*) aggregated NETs (*agg*NETs) (21–23). *Diff*NETs are formed by a complex of extracellular decondensed chromatin decorated with antimicrobial proteins and present a globular and compact shape, ranging between 25-28 µm diameter (23). S*pr*NETs are smooth, elongated web-like structures of decondensed chromatin and antimicrobial proteins, characterized by 15-17 nm in diameter thin fibers (23). Lastly, *agg*NETs consist of extracellular chromatin decorated with granular proteins originated from many neutrophils that form large clusters of NET structures measuring over 50 µm in diameter (21–23). Usually, *spr*- and *agg*NETs are the phenotypes associated with the entrapment of pathogens, such as bacteria, virus, fungi, and parasites (20, 21, 24).

Protozoan and metazoan parasite species were already described as potent NET inducers (25–28), including different stages of the closely related *F. hepatica* (16, 29), *Fasciola gigantica* (30), *Schistosoma japonicum* (31) among other trematodes (31–33). Hence, aim of this study is to describe the innate immune responses of bovine neutrophils to the trematode *C. daubneyi* via visual and molecular characterization of these host-parasite interactions. Analyses of scanning electron microscopy (SEM), immunofluorescence, 3D-holotomographic microscopic live cell imaging, oxygen consumption- and extracellular acidification rates, ROS production and ATP concentration confirmed *C. daubneyi* as weak inducer of bovine neutrophil activation. Our data represent ground investigations on the understanding of *C. daubneyi*-mediated host innate immune reactions of exposed bovine neutrophils thereby contributing to comprehend early parasite-host interactions during retrograde NEJ intestinal submucosal migration before the establishment of adult ruminal infection.

## 2 Materials and methods

### 2.1 Ethic statement

This study was performed in accordance with the Justus Liebig University Giessen Animal Care Committee Guidelines. Protocols were approved by the Ethics Commission for Experimental Animal Studies of the Federal State of Hesse (Regierungspräsidium Giessen; GI 18/10 Nr. V 2/2022; JLU-No. 0002_V) and are in accordance with European Animal Welfare Legislation: ART13TFEU and currently applicable German Animal Protection Laws.

### 2.2 Bovine neutrophil isolation

Healthy adult dairy cows (*n* = 3-6) were bled by puncturing the jugular vein, and peripheral blood was collected in heparinized sterile plastic tubes (Kabe Labortechnik). Further, 20 mL of heparinized blood was diluted in 20 mL sterile PBS with 0.02% EDTA (ethylenediaminetetraacetic acid, Carl Roth), carefully layered on top of 12 ml Histopaque-1077 separating solution (density = 1.077 g/L; 10771, Sigma-Aldrich) and centrifuged (800 × *g*, 45 min) without brake. After removal of plasma and peripheral blood mononuclear cells (PBMC), the cell pellet was suspended in 20 mL of lysis buffer (5.5 mM NaH_2_PO_4_, 10.8 mM KH_2_PO_4_, and pH 7.2) and gently mixed for 60 s to lyse erythrocytes. Osmolarity was then restored with 10 mL hypertonic buffer (462 mM NaCl, 5.5 mM NaH_2_PO4, 10.8 mM KH_2_PO_4_, and pH 7.2) and 10 mL of Hank’s balanced salt solution (14065-049, Gibco). The lysis step was repeated twice until no erythrocytes were visible. Bovine neutrophils were then suspended in 5 mL of HBSS, counted in a Neubauer chamber, and allowed to rest on ice for 30 min prior to any experimental use (34).

### 2.3 Parasites

Rumen samples of naturally *C. daubneyi*-infected cattle (two 14-month old animals) were collected from a local butchery and immediately transported at 4°C to the Institute of Parasitology, Justus Liebig University Giessen. After arrival, *C. daubneyi* adults were immediately collected, washed twice in sterile PBS 1X and frozen at -20°C until further use.

Rumen fluke eggs were recovered from frozen adults. Briefly, five adult parasites were incubated in PBS overnight in a water bath at 37°C. Then, after agitation, the solution was allowed to sediment for 3 min thrice. The remaining pellet was filtered with a 100 µm filter (PluriSelect), followed by centrifugation (400 × *g*, 5 min). Eggs were resuspended in HBSS, counted and conserved at 4°C until confrontation with bovine neutrophils.

### 2.4 Molecular analyses of rumen flukes

To identify cattle paramphistomes sequence data of ribosomal DNA region (ITS2) and complete mitochondrial cytochrome oxidase subunit 1 gene (*cox1*) were generated and compared to GenBank entries. Genomic DNA was isolated from five adult flukes using DNeasy blood and tissue Kit (Qiagen), following the manufacturer´s instructions. The rDNA region was amplified using primer combinations ITS2-F and ITS2-R (35). The *cox1* gene was amplified using the primer combination JB3 (nematode COI)/trem.cox1rrnl (trematode mitochondrial rrnl reverse) (36–38). PCRs were performed in 50 μL reactions using HOT FIREPol Blend Master Mix 7.5 mM MgCl_2_ (Solis BioDyne), 200 nM of forward and reverse primers each, and 100 ng of flukes DNA under the following conditions: 2 min 94°C initial denaturation, 35 cycles 30 s 94°C, 30 s 53°C, 45 s 72°C and 5 min 72°C final extension. Amplicons were gel-purified, cloned, and sequenced by an external service provider (LGC Genomics GmbH). Complete sequences of rDNA and mitochondrial region were assembled from overlapping amplicons and analyzed by BLAST search against GenBank database. ITS2 sequences are available under accession number PQ821198-PQ821199.

### 2.5 Soluble *Calicophoron daubneyi* antigen preparation

For soluble *C. daubneyi* antigen (*Cd*Ag) preparation, 6 adult flukes were frozen in liquid nitrogen and grounded in a previously UV-sterilized and cooled mortar (-80°C overnight) on ice. Then, 400 μL sterile PBS 1X supplemented with protease inhibitor cocktail (1:200; Sigma-Aldrich) were added into the mortar and collected. The suspension was then sonicated in ice bath with a Sonorex Super RK31^®^ bath-type sonicator (Bandelin) for 5 cycles of 15 s. The sonicated material was centrifuged at 10,000 × *g* for 20 min at 4°C. The protein concentration of the supernatant was measured using the Pierce™ BCA Protein Assay Kit (Thermo Scientific™) and final *Cd*Ag solutions were stored aliquoted at -80°C until further use.

### 2.6 Visualization of *Calicophoron daubneyi*-induced cellular immune responses

#### 2.6.1 Scanning electron microscopy analysis

Bovine neutrophils (*n* = 3; 2 ×10^5^ neutrophils) were allowed to settle on 10 mm coverslips (Thermo Fisher Scientific) pre-coated with 0.01% poly-_L_-lysine (Sigma-Aldrich) before being stimulated with either *Cd*Ag (100 µg/mL), *C. daubneyi* eggs (5-10 eggs per slide), or plain medium as negative control, and incubated for 180 min at 37°C and 5% CO_2_. After incubation, cells were fixed with 2.5% glutaraldehyde (Merk), post-fixed in 1% osmium tetroxide (Merk), washed with distilled water, dehydrated, critical point dried by CO_2_ treatment, and sputtered with gold particles. Samples were analyzed with a Philips XL30^®^ scanning electron microscope at the Institute of Anatomy and Cell Biology, Justus Liebig University Giessen, Germany.

#### 2.6.2 Immunofluorescence microscopy analysis

Bovine neutrophils (*n* = 3; 2 ×10^5^ neutrophils) were co-cultured with *Cd*Ag 100 µg/mL (37°C, 5% CO_2_, 120 min) on fibronectin- (2.5 µg/mL) pretreated coverslips (15 mm diameter, Thermo Fisher Scientific), fixed in 4% paraformaldehyde (Merck), and stored at 4°C until further use as previously described (16). For NET visualization, anti-histones (MAB3422, Chemico Int; 1:200) and anti-NE (neutrophil elastase; AB68672, Abcam; 1:200) antibodies were used to detect the respective proteins on NET structures. To stain DNA, DAPI (Fluoromount G™ Mounting Medium, Invitrogen™) was used. For antibody related reactions, fixed samples were washed three times with PBS and incubated in corresponding primary antibody solutions (4 °C, overnight). After three washings in PBS, samples were incubated in secondary antibody solutions [goat anti-mouse Alexa Fluor 594 and goat anti-rabbit Alexa Fluor 488 (Thermo Fischer Scientific A-11005 and A-11008), both 1:500 dissolved in buffer (PBS 1X, 3% BSA, 0.3 Triton X-100)] for 1 h at RT in the dark. Finally, samples were washed thrice in PBS and mounted in an anti-fading buffer (Fluoromount G™ Mounting Medium, Invitrogen™), and allowed to settle for 24 h at room temperature (RT) prior to visualization with an inverted Olympus IX81^®^ epifluorescence microscope equipped with a XM10^®^ digital camera (Olympus). Image acquisition was performed using Olympus CellSens Imaging Software and applying identical brightness and contrast conditions within the datasets of each biological experiment.

### 2.7 Live cell imaging using 3D-holotomographic microscopy to investigate cellular innate immune responses to *Cd*Ag

Bovine neutrophils (*n* = 3; 1 × 10^6^ neutrophils) were centrifuged at 300 × *g*, 10 min, RT. Cell pellets were suspended in 2 mL of imaging medium containing 0.1% bovine serum albumin (BSA; Sigma–Aldrich) and 2 µM Hoechst 34580 (Invitrogen™). One mL of cell suspension was seeded in a 35 mm Ibidi^®^ low profile plastic cell plate and incubated in a top-stage incubation chamber (Ibidi^®^) at 37°C and 5% CO_2_ atmosphere. After 30 min, 100 µg/mL *Cd*Ag were added to the isolated bovine neutrophils. Image acquisition was set for refractive index (RI; 3D tomography) and blue channel detection, applying timelapse settings (image acquisition over 120 min) using a 3D Cell Explorer-fluo (Nanolive). At the end of the experiment, each channel was exported separately using Steve software v.1.6 (Nanolive) and managed with Image J software (Fiji version 1.7, NIH). RI holotomographic reconstruction was obtained using Z project. For the 3D reconstruction images, the software Steve (Nanolive) was used.

### 2.8 Nuclear area expansion (NAE) and spectrofluorometric-based NETosis quantification

NAE-based quantification of *C. daubneyi*-induced NETosis was performed as described elsewhere (16). Briefly, bovine neutrophils (*n* = 3; 2 ×10^5^ neutrophils) were co-cultured with *Cd*Ag 100 µg/mL or plain medium, as negative control, (120 min, 37°C, 5% CO_2_). After incubation, neutrophils were fixed in 2% paraformaldehyde (Merk), washed three times with PBS 1X, and mounted in Fluoromount G™ Mounting Medium (Invitrogen™) for 24 h at RT in the dark. Using an inverted Olympus IX81^®^ epifluorescence microscope equipped with a XM10^®^ digital camera (Olympus) and Olympus CellSens Imaging Software, five images were captured randomly for each condition and NAE of single cells was analyzed via DANA I and DANA II software. Single cells presenting decondensed nucleus and exceeding a threshold of 90 µm^2^ were considered as undergoing the process of NETosis. Over 570 neutrophils were analyzed per experimental condition, from three different blood donors.

Spectrofluorometric-based analyses was performed as classical NETosis quantification technique. Bovine neutrophils (*n* = 3; 2 ×10^5^ neutrophils) were co-cultured with *Cd*Ag 100 µg/mL, *Cd*Ag 10 µg/mL or plain medium for 120 min in 96-well plastic flat-bottom plates (Greiner), at 37°C and 5% CO_2_ atmosphere. After incubation, samples were treated with micrococcal nuclease (0.1 U/µL, New England Biolabs, 15 min, 37°C, 5% CO_2_) and later centrifuged (300 × *g*, 5 min). Supernatants were transferred into new wells and PicoGreen^®^ (50 µL/sample, Thermo Scientific, diluted 1:200 in 10 nM Tris/1 nM EDTA buffer) was added in the dark. NETosis was estimated via spectrofluorometric analyses at an excitation wavelength of 484 nm and an emission wavelength of 520 nm in an automated multiplate reader (Varioskan^®^ Flash, Thermo Scientific) (16). Bovine neutrophils in plain medium (negative control) or with zymosan (1 mg/mL, Invitrogen) as positive control were also considered.

### 2.9 *Calicophoron daubneyi* exposed bovine neutrophils degranulation assay

Given that neutrophil degranulation was observed in immunofluorescence analyses, it was measured by means of myeloperoxidase (MPO) release, in presence of cytochalasin B (cytoB; Sigma-Aldrich) (39). For total MPO content, CTAB (cetyltrimethylammonium bromide, 0.02%, Sigma-Aldrich) was used. Medium (125 µL) containing *Cd*Ag (100 µg/mL), calcium ionophore A23187 (CaI, 5 µg/mL, Sigma-Aldrich) or CTAB (lysed neutrophils) was added to a 96-well plate (Greiner). Background wells containing only medium were added. Bovine neutrophils (*n* = 3; 1.25 × 10^6^ in 25 µL) were added to each well, including negative control and background wells (neutrophils and HBSS). The plate was incubated at 37°C for 90 min. At the end of the incubation period, 50 µL of pre-warmed 2.5 mM TMB (3,3’,5,5’-tetramethylbenzidine hydrochloride, Sigma-Aldrich) were added to each well, and immediately followed by 50 µL of pre-warmed 5 mM H_2_O_2_ (Roth). The reaction was allowed to develop for 2 min than 50 µL of 4 M sulphuric acid (Sigma-Aldrich) were added to each well to stop the reaction. The plate was centrifuged (600 × *g*, 10 min) and 200 µL of supernatant were transferred to a new flat-bottom 96-well plate (Greiner). Optical density (OD) was determined at 405 nm in an automated multiplate reader (Varioskan^®^ Flash, Thermo Scientific). Reactions were tested in duplicates of three biological replicates. The percentage of MPO release was calculated by the following formula:


$$\text{\% MPO release}=\frac{\left(\text{OD stimulated}-\text{OD background}\right)}{\left(\text{OD lysed}-\text{OD background}\right)}\;\times \;100$$

### 2.10 Quantification of neutrophils oxygen consumption rate and extracellular acidification rate following *Cd*Ag stimulation

Activation of bovine neutrophils was monitored using the Seahorse XF analyzer (Agilent). A total of 1×10^6^ bovine neutrophils from three blood donors were pelleted [500 × *g*, 10 min, RT]. Cell pellets were re-suspended in 250 µL of XF assay medium (Agilent) supplemented with 2 mM of L-glutamine, 1 mM pyruvate, and 10 mM glucose. A total of 2×10^5^ cells were gently placed in each well of an eight-well XF analyzer plate (Agilent) pre-coated with 0.001% poly-_L_-lysine (Sigma-Aldrich) for 30 min. Later, the XF assay medium (Agilent) was adjusted to 180 ml total volume per well and cells were incubated at 37°C without CO_2_ supplementation for 45 min before Seahorse XF measurements. *Cd*Ag (0.01-100 µg/mL) were suspended in XF assay medium (final volume 20 µL) and supplemented to the cells via instrument-own injection ports after baseline measurements. Plain XF assay medium served as negative control. The total assay duration was 200-240 min. Background subtraction and determination of OCR/ECAR registries, as well as area under the curve (AUC) calculations were performed by using the Seahorse Agilent analytics software Wave^®^ (Desktop Version, Agilent) and GraphPad Prism^®^ versions 8.3.1 for Windows 10, GraphPad^®^ Software, San Diego, California USA.

### 2.11 Intra- and extracellular ROS production in *Cd*Ag-stimulated bovine neutrophils

Intracellular ROS production of bovine neutrophils was assessed by oxidation of 2′,7′-dichlorofluorescein diacetate (DCFH-DA, Sigma-Aldrich) to fluorescent DCF following previous reports (40, 41). Freshly isolated neutrophils (*n* = 3) were resuspended in sterile 1X HBSS containing Ca^2+^ and incubated with soluble *Cd*Ag (10 or 100 μg/mL) (4 ×10^5^ cells/well; 37°C, 30 min, in duplicates), final volume of the reaction 200 μL. Afterwards, DCFH-DA (10 μg/mL) was added to each duplicate. For positive control zymosan was used (final concentration 1 mg/mL (42);). The relative fluorescence units (RFU) were detected every 15 min for a period of 120 min applying 485 nm excitation and 530 nm emission wavelengths (Varioskan^®^ Flash, Thermo Scientific).

Amplex Red^®^ reagent (Invitrogen) was used to assay extracellular ROS production. Bovine neutrophils (2 ×10^5^ cells in 100 μL; *n* = 3, in duplicates) were seeded in a plastic 96-well plate (Greiner). Pre-warmed (66.6 µL, 37°C, 10 min) Amplex Red^®^ reaction mixture [37.5 μL Amplex Red^®^ (20 mM) and 75 μL horseradish peroxidase (HRP; 20 U/mL, Merk), in 5.9 mL RPMI 1640 medium without phenol red (Sigma-Aldrich)] were added to each well. Immediately before starting measurements, the stimuli were added in 33.5 μL [10 or 100 µg/mL *Cd*Ag; zymosan (1 mg/mL) or plain medium]. Fluorescence intensity was measured in an automated multiplate reader (Varioskan^®^ Flash, Thermo Scientific) at 530 nm excitation wavelength and 590 nm emission wavelength (43) at determined time points (0, 15, 30, 60, 90, 120 and 150 min of stimulation) and being incubated (37°C, 5% CO_2_) between measurements.

### 2.12 Measurement of total and extracellular ATP concentration after *Cd*Ag stimulation

Bovine neutrophils (*n* = 3; 1×10^6^ cells) from three healthy donors were co-cultured with *Cd*Ag (10 - 100 µg/mL) in HBSS 1X (14065-049; Gibco) for 15 min (37°C, 5% CO_2_). Cells were stimulated with phorbol myristate acetate (PMA)/ionomycin (100 nM/5 M; Sigma-Aldrich) as a positive control. After resting on ice for 5 min, cells were centrifuged at 600 × *g* for 5 min and extracellular adenosine triphosphate (ATP) concentrations were determined in supernatants using an ATP Determination kit (A22066; Invitrogen) according to manufacturer’s instructions. Total ATP concentration was quantified in whole cell pellets using CellTiter-Glo luminescent. All samples were analyzed by luminometry with an automated reader (Luminoskan Flash).

### 2.13 Statistical analysis

Statistical analyses and all graphs (mean ± SD) were performed using GraphPad Prism^®^ versions 8.3.1-9.2.0 for Windows 10, GraphPad^®^ Software, San Diego, California USA, www.graphpad.com. For comparison of two groups, unpaired two-tailed Mann-Whitney tests were applied, and for comparing three or more groups, no pairing non-parametric Kruskal-Wallis test was performed with Dunn’s multiple comparison test. For ATP analyses ordinary one-way ANOVA and Dunnett’s test for multiple comparisons were applied. Statistical significance was defined at *p* < 0.05.

## 3 Results

### 3.1 *Calicophoron daubneyi* induce bovine NETosis

**Figure 2:**
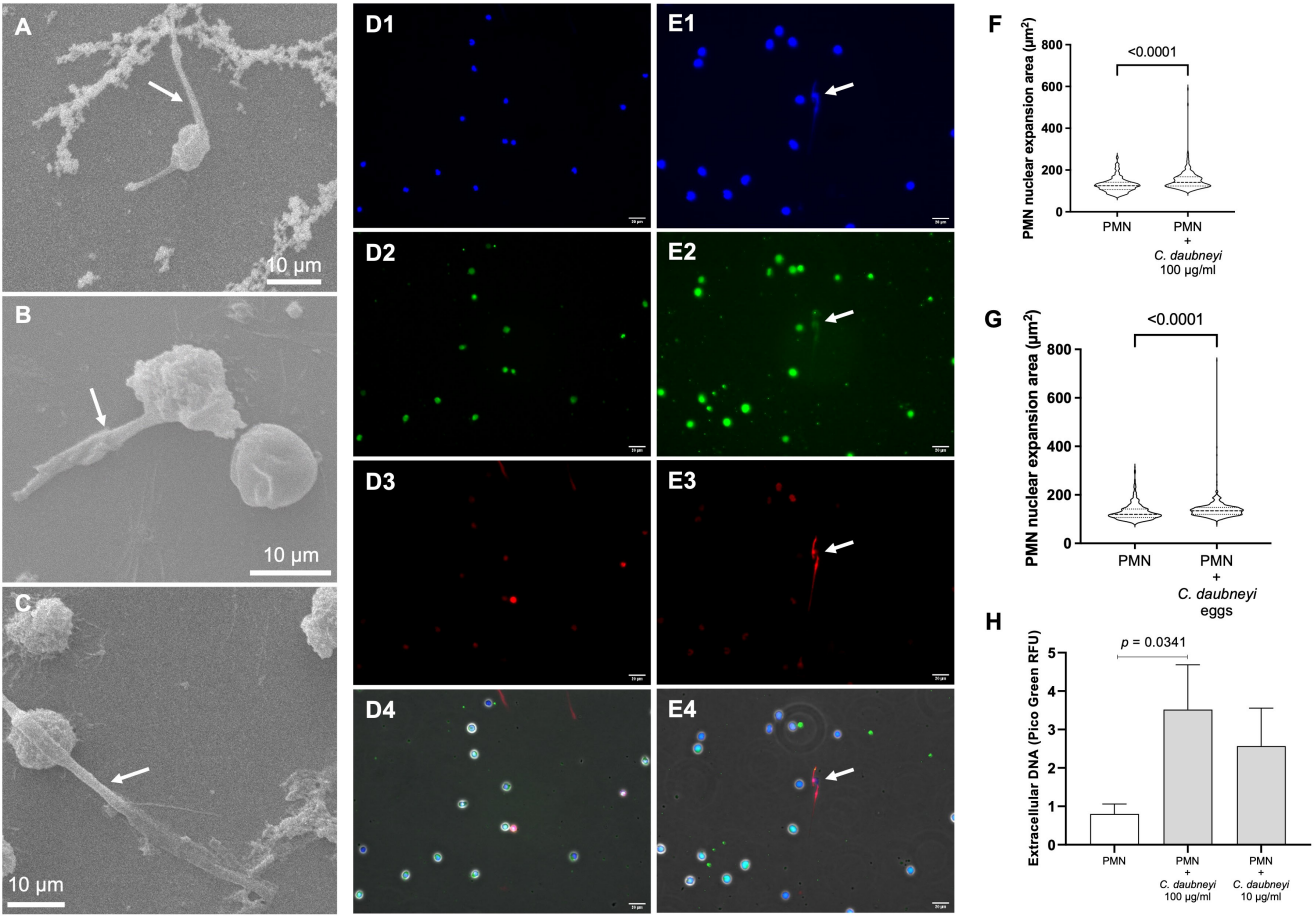
*Calicophoron daubneyi* exposure induce weak bovine NETosis. **(A-C)** SEM analyses confirm bovine neutrophils cast short spread NETs (*spr*NETs, arrows) when co-cultured with *C. daubneyi* soluble antigen (*Cd*Ag 100 µg/mL). Non-activated neutrophils preserve their characteristic globular shape. Scale bar 10 µm. **(D, E)** Non-stimulated **(D1–D4)** and stimulated **(E1–E4)** bovine neutrophils (*Cd*Ag 100 µg/m), analyzed via immunofluorescence, identify classical NETs components such as extracellular DNA (blue, **D1**, **E1**), neutrophil elastase (green, **D2**, **E2**), and histones (red, **D3**, **E3**). Stimulated neutrophils cast short *spr*NETs (merge, **E4**) which co-localize all NETs components, while non-stimulated neutrophils remain unaltered (merge, **D4**). Scale bar 20 µm. **(F)** Polymorphonuclear neutrophil (PMN) nuclear area expansion (NAE, µm^2^) is significantly increased in *Cd*Ag-stimulated neutrophils (*n* = 3, *p* < 0.0001) when compared to non-stimulated controls. **(G)** *C. daubneyi* eggs induce significant enhancement of NAE (*n* = 3, *p* < 0.0001). **(H)** Extracellular spectrofluorometric DNA-based quantification of NETosis via PicoGreen^®^-derived fluorescence intensities after 120 min co-culture of bovine neutrophils and *Cd*Ag 100 µg/mL results in significantly higher amounts of extracellular DNA than negative control (PMN alone) (*n* = 3, *p* = 0.0341).

Following genetic analysis confirmation of rumen flukes as *Calicophoron daubneyi* specimens (**Supplementary Table 1**), *C. daubneyi*-induced NETosis was analyzed via different methods, i.e., SEM, immunofluorescence, nuclear area expansion (NAE) and spectrofluorometric quantification. SEM analyses revealed that bovine neutrophils co-cultured with 100 µg/mL *Cd*Ag extruded short length NET filaments (**Figures 2A–C**, arrows), i.e., short spread-like NET structures (*spr*NETs), while preserving their characteristic round configuration. Other neutrophils remain unchanged (**Figures 2B, C**). Immunofluorescence analyses of co-cultured bovine neutrophils with C*d*Ag 100 µg/mL confirmed the presence of classical components of NETs (**Figures 2D, E**) in short *spr*NETs, while no extruded NET filaments were visible in non-exposed controls (**Figures 2D2–4**). Co-localization of extracellular DNA (**Figure 2E1**, blue), neutrophil elastase (**Figure 2E2**, green) and histone (H1-4, **Figure 2E3**, red) were identified in *Cd*Ag-induced NETosis (**Figure 2E4**). Also, early NETosis was studied via NAE analyses, as one of the earliest events of NET formation is the decondensation of neutrophil nuclear chromatin. After co-culture of *Cd*Ag (100 µg/mL) or *Cd*-eggs with bovine neutrophils, exposed neutrophils presented a significant increase in NAE when compared to non-exposed controls (**Figures 2F, G**, *p* < 0.0001). In total, more than 570 neutrophils were analyzed individually per condition (*n* = 3). Additionally, extracellular spectrofluorometric DNA-based quantification of NETosis via PicoGreen^®^-derived fluorescence intensities was measured after 120 min co-culture of bovine neutrophils and *Cd*Ag 10 and 100 µg/mL (**Figure 2H**). In both cases a rise in PicoGreen^®^ RFU was observed when compared to neutrophils alone. However, only *Cd*Ag 100 µg/mL concentration revealed to be significantly higher (**Figure 2H**, *p* = 0.0341). All these results confirm that *C. daubneyi* is weak inducer of bovine NETosis.

### 3.2 Degranulation and chemotaxis of bovine neutrophils in presence of *Calicophoron daubneyi* antigens

**Figure 3:**
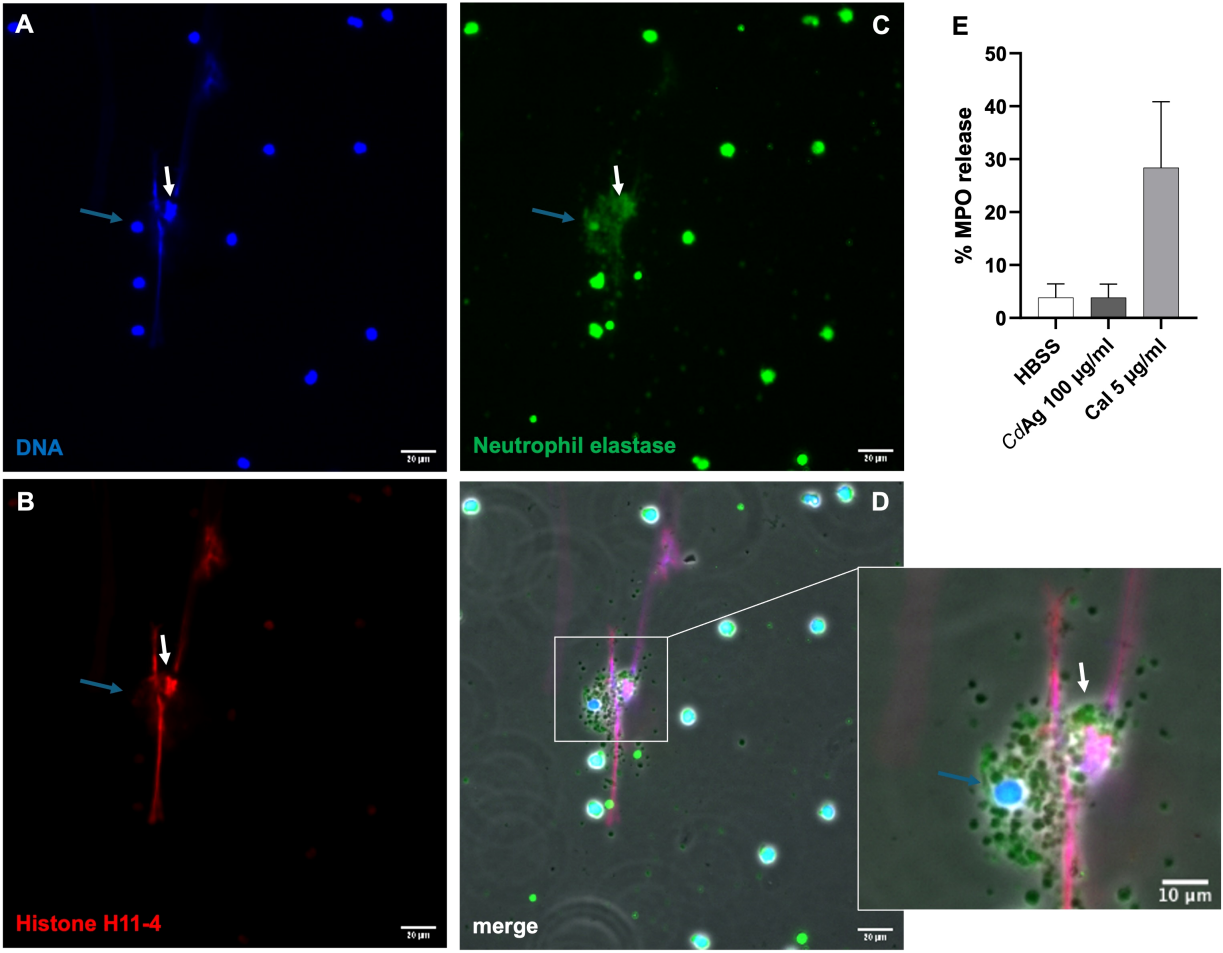
Bovine neutrophils stimulated with *Calicophoron daubneyi* antigen (*Cd*Ag100 µg/mL) perform NETosis and degranulation. Immunofluorescence analyses confirm classical NETs components on short *spr*NETs (white arrows): **(A)** extracellular DNA (blue), **(B)** neutrophil elastase (green), **(C)** histones (red), and simultaneous accumulation of extracellular granules (blue arrow) compatible to degranulation events **(D)** merge – zoom. Scale bar 20 µm. **(E)** Exposure to *Cd*Ag 100 µg/mL does not significantly increase the release of myeloperoxidase (MPO), contrary to the positive control (CaI 5 µg/mL) (*n* = 3).

**Figure 4:**
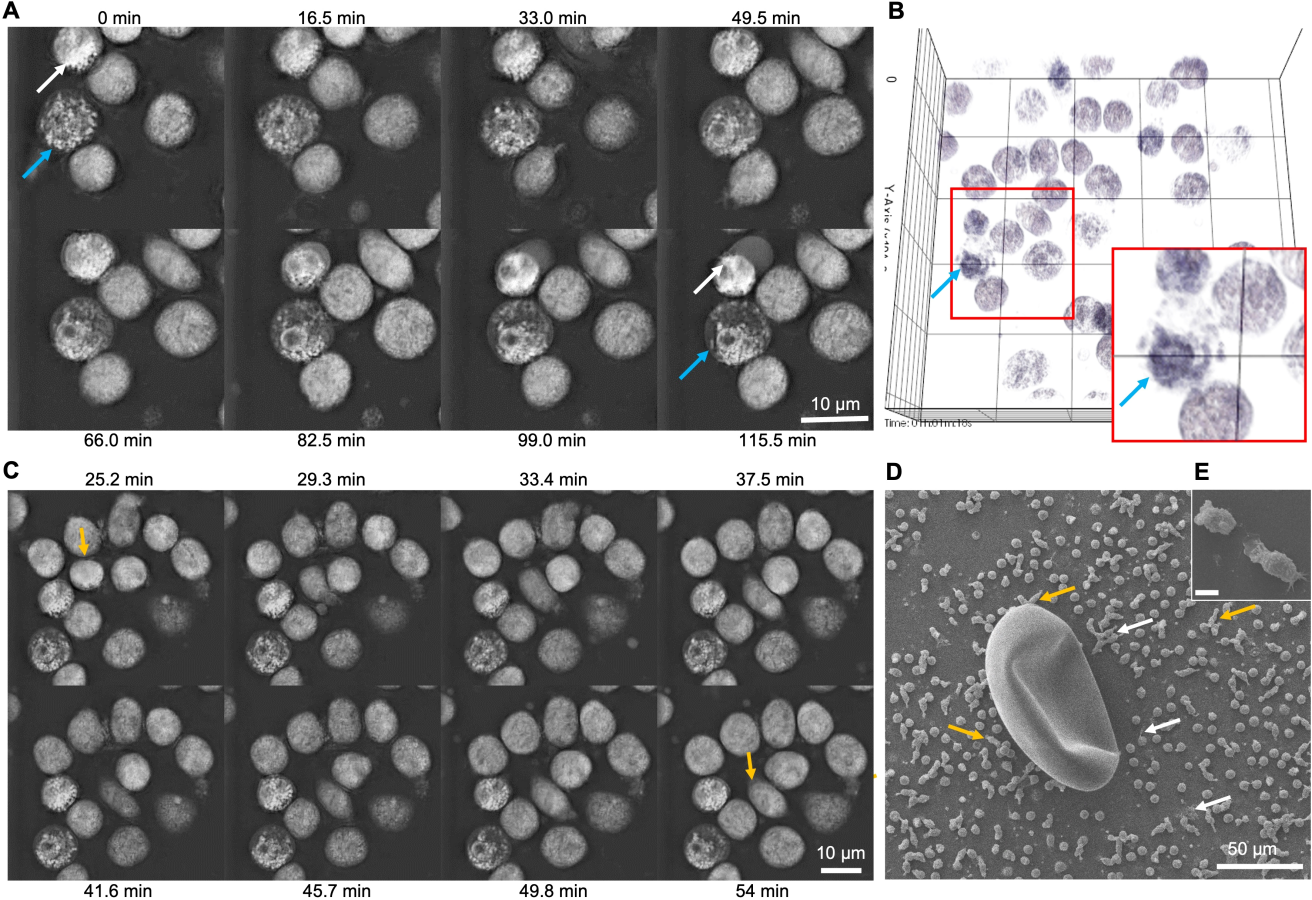
Live 3D-holotomographic microscopy analyses of bovine neutrophils exposed to *Calicophoron daubneyi* unveil degranulation and chemotaxis events. **(A)** *Calicophoron daubneyi* antigen (100 µg/mL, CdAg) induced degranulation of neutrophils (arrows) as early as 5 min incubation until the end of the experiment. **(B)** 3D reconstruction and digital staining of holotomographic images confirm shift and accumulation of granules at one side of the neutrophil (zoom, blue arrow). **(C)** Chemotactic events are recorded between 25 and 54 min of incubation (yellow arrows) with displacement of stimulated neutrophils with CdAg 100 µg/mL. **(D)** Chemotaxis can be observed after co-culture of neutrophils with *Calicophoron daubneyi* eggs and analyzed via SEM. Neutrophils are elongated and moving in the direction of the eggs (yellow arrows), while other neutrophils are activated and cast short *spr*NETs (white arrows) in the vicinity of the eggs. **(E)** Elongated neutrophils in detail. Rupture is an artefact caused by the SEM analyses.

Immunofluorescence analyses of *Cd*Ag-exposed bovine neutrophils (*n* = 3) unveiled not only the formation of short *spr*NETs but also granular structures similar to the observed in degranulation process of stimulated neutrophils (**Figures 3A–D**). During neutrophil activation and degranulation, cytoplasmatic granules fuse with the cell membrane thereby discharging their cargo. Here, we observed small round granules in the vicinity of activated neutrophils casting NETs (**Figure 3D**), resembling discharged granules, which led to further investigations. Therefore, as degranulation assay, the myeloperoxidase (MPO) release test was performed showing no alteration on the percentage of MPO release on confronted bovine neutrophils (**Figure 3E**). Noteworthy, during live cell 3D-holotomographic microscopy analyses of bovine neutrophils stimulated with *Cd*Ag 100 µg/mL similar degranulation images were observed within activated neutrophils (**Figures 4A, B**, arrows). As early as 30 min after stimulation of bovine neutrophils with *Cd*Ag 100 µg/mL, it was observed a displacement of neutrophils within the microscopic field (**Figure 4C**, yellow arrow). Displacements were also observed via SEM analyses of bovine neutrophils co-cultured with *C. daubneyi-*eggs (**Figures 4D, E**, yellow arrows), confirming that *C. daubneyi* is capable of bovine neutrophil activation.

### 3.3 Exposure to *Calicophoron daubneyi* antigen induces oxidative responses of bovine neutrophils and extracellular acidification

**Figure 5:**
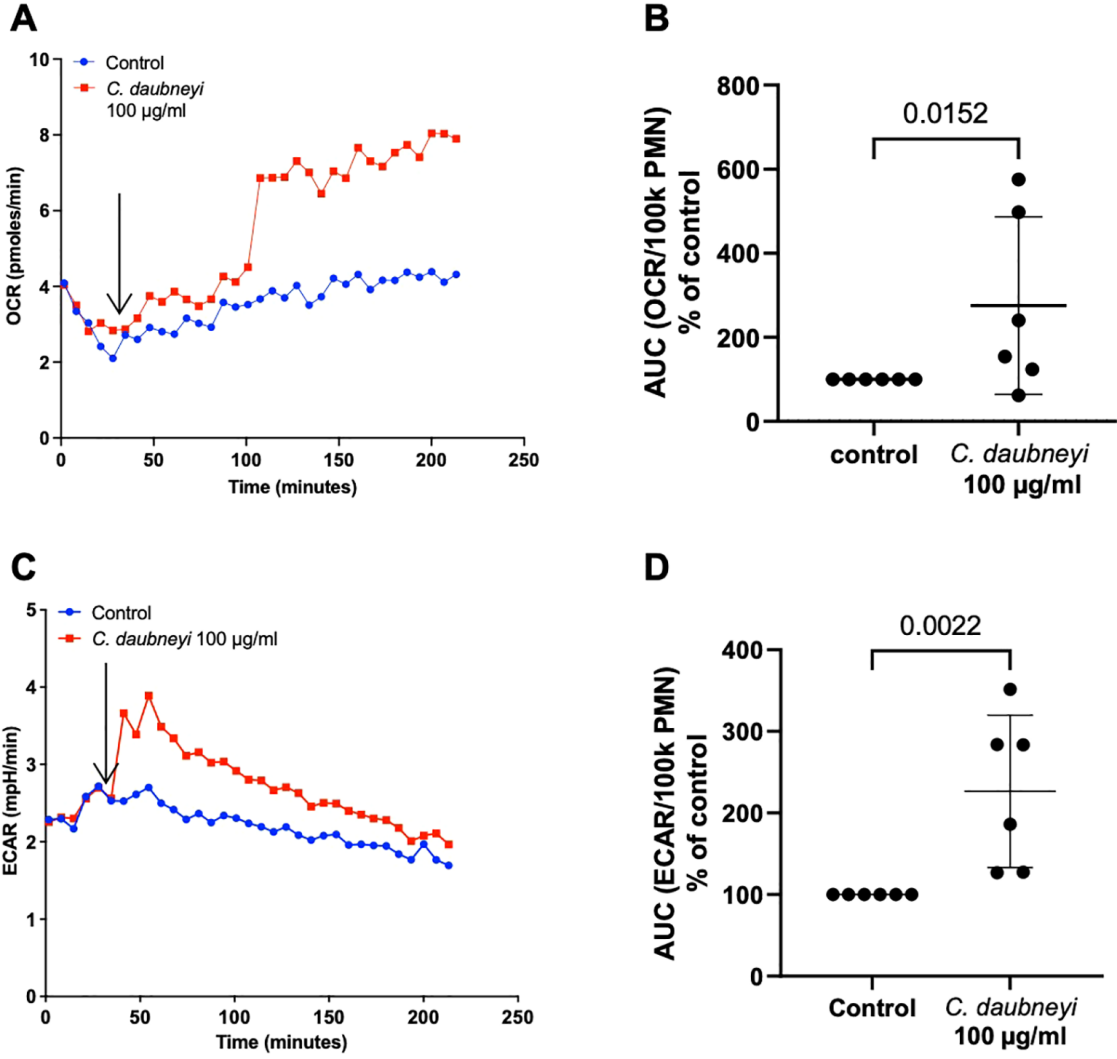
Exposure of bovine neutrophils to *Calicophoron daubneyi* antigen (*Cd*Ag 100 µg/mL) induces oxidative responses and extracellular acidification. **(A)** Oxygen consumption rates (OCR) remained unaltered after injection of *Cd*Ag into the wells, after 5 baseline measurements (arrow), but increased suddenly at 100 min of incubation and until the end of the experiment (*n* = 6, mean). **(B)** AUC of OCR is significantly higher in exposed neutrophils, even with strong individual variability (*n* = 6, *p* = 0.0152). **(C)** Extracellular acidification rates (ECAR) show marked increase (*n* = 6, mean) immediately following injection of *Cd*Ag 100 µg/mL into the wells, after 5 baseline measurements (arrow). **(D)** AUC of ECAR in stimulated neutrophils is significantly higher than controls, with three of the animals reacting strongly (*n* = 6, *p* = 0.0022).

Oxygen consumption rate (OCR) measurements reflect neutrophil oxidative responses due to oxidative burst activity by assessing NADPH oxidase (NOX)-related oxygen consumption and reflects mitochondrial respiratory activity, while extracellular acidification rates (ECAR) reflect production and release of lactate as a product of glycolysis. The AUC (area under the curve) analyses revealed *Cd*Ag 100 µg/mL induced increase of OCR (*p* = 0.0152, *n* = 6) and ECAR (*p* = 0.0022, *n* = 6) levels on stimulated bovine neutrophils when compared to controls (**Figures 5A–D**). OCR increase is detected after 100 min of experiment which might be related to glycolysis involvement. High variability in cellular immune responses between individual bovine donors could be observed. Here, only three out of the six individuals responded strongly upon antigen stimulation. The same experiments with *Cd*Ag 10 µg/mL were performed but no significant changes were reported, as for lower concentrations (**Supplementary Figure 1**).

### 3.4 Intra- and extracellular ROS production is not altered by *Calicophoron daubneyi* antigen exposure

**Figure 6:**
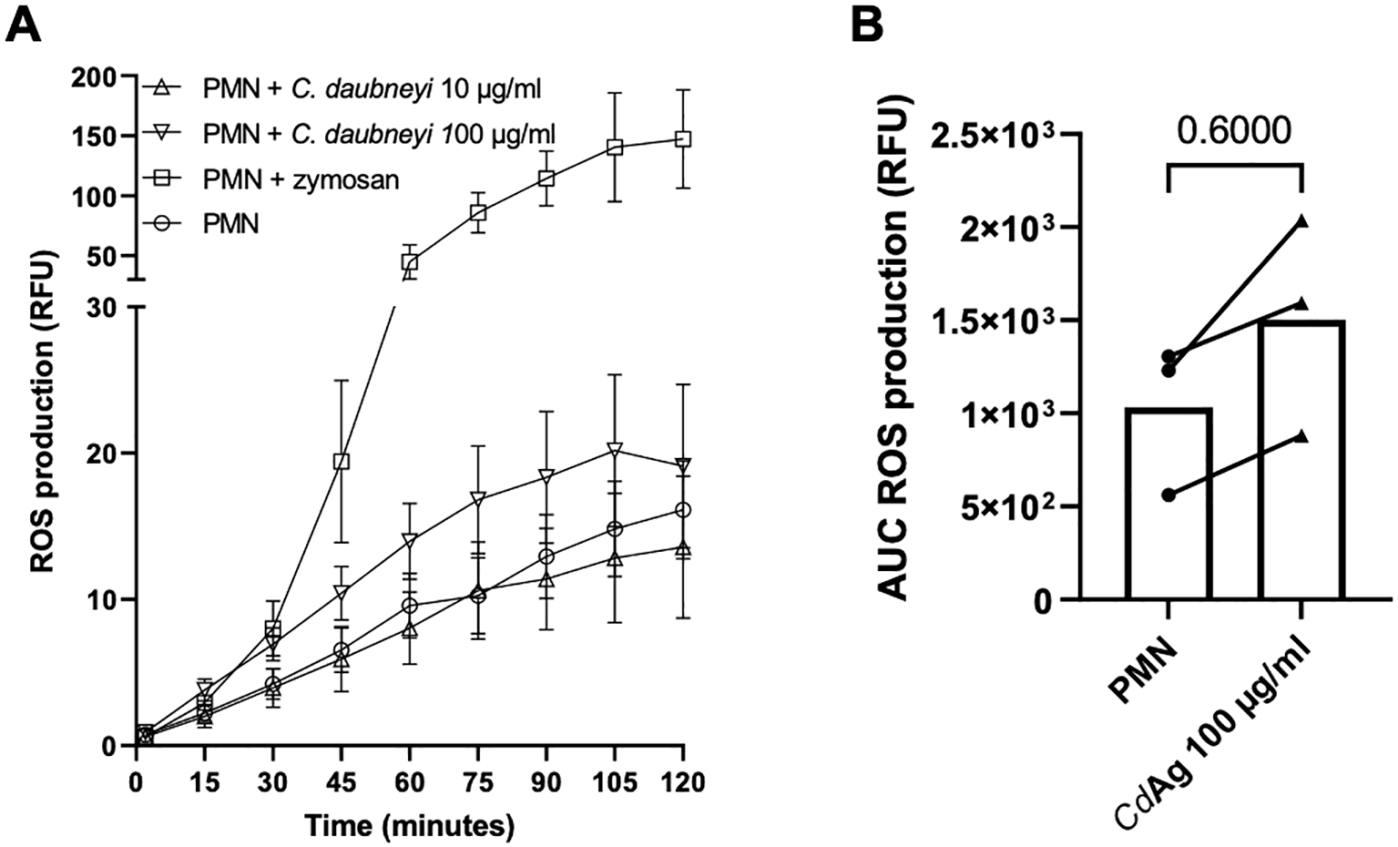
*Calicophoron daubneyi* fails to induce ROS production in bovine neutrophils. **(A)** ROS production was measured for 120 min of incubation. *C*. *daubneyi* antigen 100 µg/mL induced a slight increase in ROS production. **(B)** Differences of ROS production of exposed and non-exposed neutrophils are not significant (*n* = 3, *p* = 0.600).

Intracellular ROS production (*n* = 3) was measured by oxidation of 2′,7′-dichlorofluorescein diacetate to fluorescent DCF (**Figure 6A**). During 120 min, ROS production was only slightly increased by stimulation of bovine neutrophils with *Cd*Ag 100 µg/mL (**Figure 6B**, *p* = 0.600), but *Cd*Ag 10 µg/mL failed to induce neutrophil-derived ROS production (**Figure 6A**), indicating a dose dependent induction of intracellular ROS production. Zymosan, a well-known inducer of neutrophil respiratory burst activity, was used as positive control. For extracellular ROS production, the highly specific and sensitive Amplex Red quantification assay was performed to detect extracellular hydrogen peroxide (H_2_O_2_). The H_2_O_2_ production was not altered by stimulation with *Cd*Ag 10 µg/mL over time (**Supplementary Figure 2**). *Cd*Ag 100 µg/mL induced a change in H_2_O_2_ production, even higher than zymosan effect (**Supplementary Figure 2**). However, the same magnitude of effect was observed when *Cd*Ag 100 µg/mL alone was tested, showing that *Cd*Ag 100 µg/mL is also not capable of inducing any H_2_O_2_ production. These results show that *C. daubneyi* antigen does not induce ROS production, commonly associated with mammalian NET formation.

### 3.5 *Calicophoron daubneyi* antigen alters total ATP concentration in exposed bovine neutrophils

**Figure 7:**
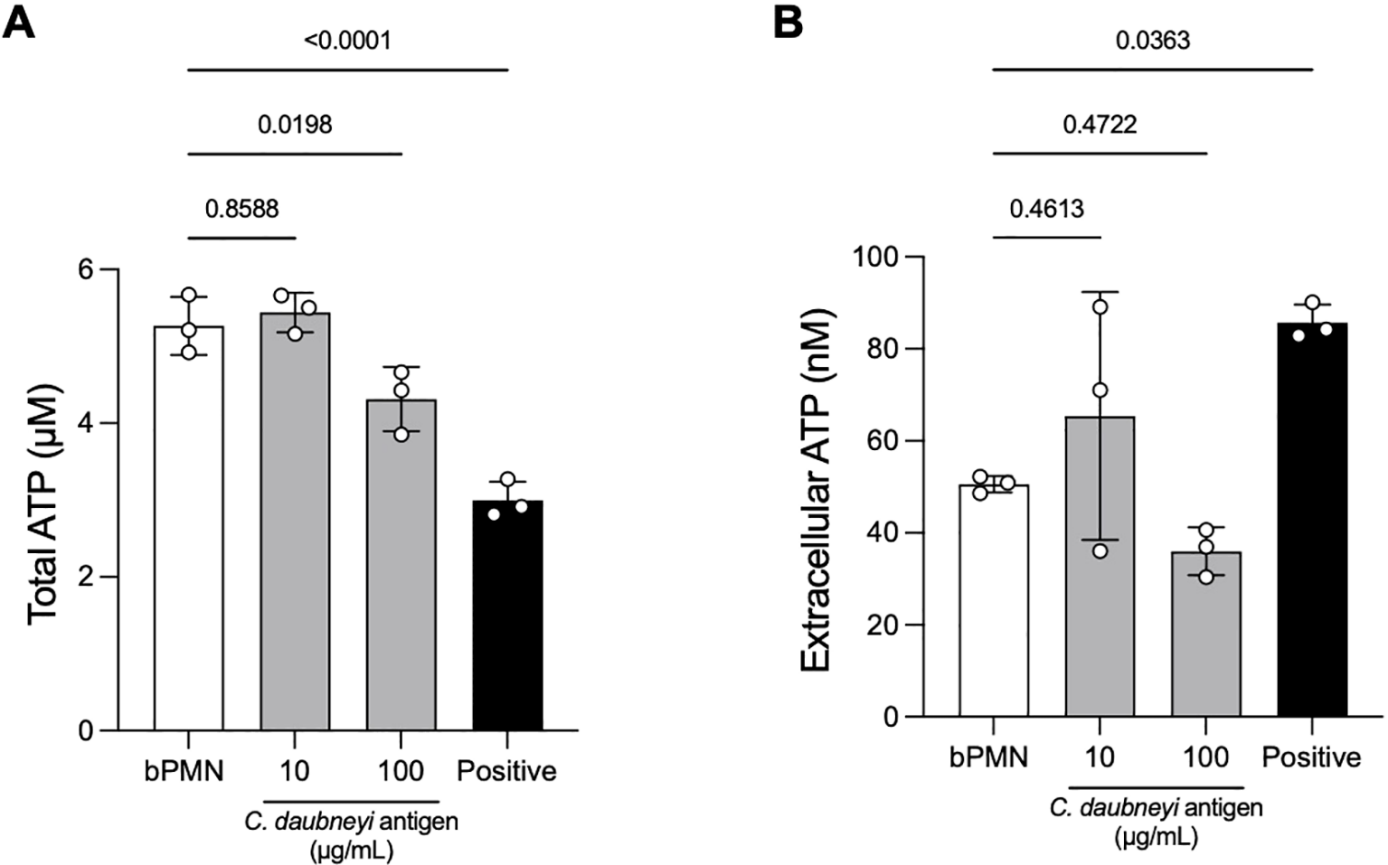
*Calicophoron daubneyi* decreased total ATP concentrations of stimulated neutrophils. **(A)** Total ATP concentrations (µM) of bovine neutrophils exposed to *Calicophoron daubneyi* antigen (*Cd*Ag) 100 µg/mL are significantly lower than negative control (bPMN, *n* = 3, *p* = 0.0198), in line with positive control results (PMA/ionomycin, *p* < 0.0001). **(B)** *Cd*Ag 100 µg/mL fails to increase extracellular ATP concentrations (nM), similarly, to lower *Cd*Ag concentrations (10 µg/mL), and contrary to positive control (PMA/ionomycin, *p* = 0.0363).

To investigate the effects of *Cd*Ag on total- and extracellular ATP concentrations of stimulated neutrophils (*n* = 3), ATP concentrations were quantified by luminometry at 15 min of stimulation (**Figure 7**). For positive controls, PMA/ionomycin were used. Upon *Cd*Ag 10 µg/mL stimulation, both total and extracellular ATP measurements showed no differences between stimulated and non-stimulated neutrophils (**Figure 7**). With *Cd*Ag 100 µg/mL, total ATP concentration was significantly lower than negative control (**Figure 7A**, *p* = 0.0198), while ATP levels measured on supernatants remained unaltered (**Figure 7B**, *p* = 0.4722). Nevertheless, bovine neutrophils stimulated with PMA and ionomycin showed a significant drop in ATP levels (**Figure 7A**, *p* < 0.0001) related to ATP consumption, and an increasing extracellular ATP concentration (**Figure 7B**, *p* = 0.0363).

## 4 Discussion

*Calicophoron daubneyi* paramphistomosis has recently been increasing across Europe and, in some geographical areas, it is even more prevalent than *F. hepatica* (8). Adult stages typically inhabit the rumen and reticulum of cattle, sheep and other ruminants, causing minor (i.e. rumenitis, abomasitis, papillae atrophy) or no pathogenic effects (8, 11, 13). Nevertheless, acute clinical paramphistomosis is observed when grazing animals (i. e. young cattle or sheep of any age) ingest numerous *C. daubneyi* metacercariae, which excyst simultaneously in the duodenum (13). Newly excysted juveniles (NEJ) colonization of the intestinal submucosa and subsequent feeding on host tissues can last up to three months, and result in substantial tissue damage (10), before completing retrograde mucosal migration to the rumen and maturation into adult forms. Currently, knowledge on *C. daubneyi* NEJ tissue-invasion strategy and associated molecular mechanisms, virulence factors and evasion of host immune response to establish new infections in the host duodenal submucosa are still scarce (9, 10).

Immediately after excystation in the duodenum, NEJ must adapt to the intestinal environment and to the expected attack by the host immune cells. As the most abundant leukocytes and the first immune cells to reach sites of infection, neutrophils are recognized as one of the most decisive cells in the development of host innate- and adaptative immune responses (18). Therefore, this study focuses, for the first time, on the ability of the rumen trematode *C. daubneyi* to induce early innate immune responses of exposed bovine neutrophils, since *C. daubneyi* stages may be confronted to neutrophils leaking into the intestinal lumen to fight metacercariae and NEJ, infiltrating submucosa and/or other tissues where NEJ migrate through or in the rumen to combat adults *in vivo*. Overall, *C. daubneyi* soluble antigen (*Cd*Ag) seems to be a weak activator of bovine neutrophil effector mechanisms, including NETosis, oxygen consumption- and extracellular acidification rates, and total ATP concentrations.

Mammalian NET formation is characterized by consecutive morphological events: disintegration of nuclear membrane, chromatin decondensation, disappearance of plasma membrane, and extrusion of DNA-based filaments into the extracellular space (44). NETs are typically formed by extracellular decondensed chromatin filaments decorated with nuclear histones and enzymatic granular components (NE, MPO, lactoferrin, cathepsin, pentraxin, gelatinase and others). Here, *Cd*Ag induced short *spr*NETs characterized by DNA backbone decorated with histones and NE, confirmed via co-localization assays, in line to the previously reported for *F. hepatica* induced NETosis in cattle and sheep (16, 29). This data corroborates *Cd*Ag soluble antigens present adequate stimuli to trigger neutrophil-mediated immune reactions and even a more effective activation of potential pathogen-associated molecular patterns (PAMP) (29). Moreover, neutrophil NAE (µm^2^) was significantly increased after exposure to *Cd*Ag and *Cd*-eggs, indicating that early NETosis events can also be triggered by *C. daubneyi*.

SEM analyses confirmed the presence of short *spr*NETs but neither *agg*NETs nor *diff*NETs were detected. Previous reports on the close related trematode *F. hepatica-*induced NETosis described these three NETs phenotypes, along with cell free- and anchored-NETs (29). Because *Cd*Ag is a weak inducer of bovine NETosis, quantification of cell free- and anchored NETosis was not performed in this study. Standard quantification of bovine NETosis was performed by PicoGreen^®^-derived fluorescence analysis and confirmed *Cd*Ag triggered a weak reaction, similarly to the observed for both bovine and ovine neutrophils confronted with *F. hepatica* antigens (*Fh*Ag) (16, 29). However, earlier studies on NETosis induced by other trematodes, namely *Fasciola gigantica* and *Schistosoma japonicum*, reported stronger reactions triggered by such trematode parasites (30, 45), implying that the rather weak responses observed with *C. daubneyi* and *F. hepatica* are not related to their parasitic group but seeming to be rather species-specific related.

One of the most common effector mechanisms of neutrophils is degranulation of several antimicrobial peptides, including, e. g., NE, proteinase 3, cathepsin G, and MPO, which are abundantly expressed in neutrophils. Remarkably, simultaneous NET formation and degranulation events were detected in bovine neutrophils exposed to *Cd*Ag 100 µg/mL, via immunofluorescence and live cell 3D-holotomographic microscopy analyses. Comparable evidence was obtained in live cell 3D-holotomographic microscopy of ovine neutrophils exposure to *Fh*Ag, as early as 30 min of incubation (29). Moreover, previous studies on *F. hepatica* excretory/secretory (ES) products confirmed these trematode products as inducers of degranulation of mast cells (46). Degranulation occurs in hierarchical order in a stepwise process and depends on specific signaling events, such as ligation of cell surface chemotactic or phagocytic receptors or the intracellular receptor TLR9 that trigger calcium- and Hck-dependent signaling pathways, leading to actin- and microtubule reorganization for the transport and docking of azurophilic granules to the plasma membrane and consequent expansion of the fusion pore ensuring the release of granule contents (47). To further investigate degranulation induced by *Cd*Ag stimulation, a direct assay to quantity degranulation of bovine neutrophils primary granules was used (39), by measuring the release of MPO stored in azurophilic granules. Even though several events compatible with degranulation were observed upon *Cd*Ag exposure, quantification of MPO release was not altered in *Cd*Ag stimulated bovine neutrophils, when compared to non-stimulated controls.

Chemotaxis, or guided cell migration, occurs as neutrophils detect extracellular chemical gradients and are attracted to the site of infection or inflammation, for example, being the primary cells recruited during innate immune responses. In live cell 3D-holotomographic microscopy analyses of *Cd*Ag-neutrophils co-cultures, stimulated neutrophils were recorded displacing in the microscopic field, starting as early as 5 min of incubation. Additionally, *Cd*-eggs derived chemotaxis was identified in SEM analyses seen as elongated neutrophils moving towards *Cd*-eggs. These observations are in line with previous reports of *F. hepatica* eggs co-cultured with bovine neutrophils (16). However, in our case, neutrophils did not attach firmly to the surface of the *Cd*-eggs contrary to the observed in *F. hepatica* experiments.

Neutrophil oxidative burst or OCR, are usually derived from NOX-based ROS production, with little or no mitochondrial contribution (34, 48). In this study, OCR was significantly enhanced in *Cd*Ag 100 µg/mL stimulated neutrophils, but the increase occurred only after 100 min of incubation, corresponding to the peak observed in intracellular ROS production assays. On the contrary, extracellular acidification rates (ECAR) were elevated soon after the stimulation of neutrophils in a dose dependent manner (**Supplementary Figure 1**), reflecting the extracellular accumulation of lactate (48). Lactate can be actively released from activated neutrophils and glycolysis-derived lactate is linked to human NET formation (49). In OCR and ECAR assays, high individual variability was noticed, given that three of the six individuals reacted weakly to the *Cd*Ag stimuli. High variability between individual bovine donors is often reported in innate immune reactions, and even though additional donors (*n* = 6) help to dilute this effect, it is not possible to be eliminated (16, 50).

As stated, ROS production induced by *Cd*Ag 100 µg/mL peaked at 100 min but it was increased from 30 min of incubation onwards. Yet, lower concentration of *Cd*Ag (10 µg/mL) hardly induced any ROS production, resembling negative control levels. ROS production was assessed after confrontation of bovine neutrophils with *F. hepatica* antigen, with similar results to the here obtained, i. e., no induction of ROS production (16). Nevertheless, the same experiment performed in the ovine system, showed a clear increase in ROS production (29) thereby showing host species-derived differences. Other protozoan parasites, e. g. *Eimeria bovis* and *Trypanosoma brucei brucei*, confirm the role of ROS production, accompanied by oxygen consumption in parasite-induced bovine NET formation (49, 51), showing that trematode-origin trigger might not be sufficient to induce detectable ROS production and does not induce this early innate defense effector mechanism. Likewise, extracellular ROS production was not changed by stimulation of neutrophils with *Cd*Ag.

Neutrophils rely on different metabolic routes (e. g. TCA cycle, oxidative phosphorylation, fatty acid oxidation, glycolysis) to fulfil their energetic, biosynthetic and functional requirements (29, 52). Moreover, intracellular ATP is essential in almost all cells, serving both as an energy source and a signaling molecule acting as “SOS sign” or alarmin, guiding the migration and regulating other key functions of neutrophils, such as chemotaxis, adhesion, ROS production, NET formation and apoptosis (34, 53). In this context, NETosis depends on ATP-based energy supply generated *via* glycolysis for active cytoskeletal rearrangements necessary for NET extrusion (49, 54). In presence of *Cd*Ag, the total and extracellular ATP concentrations were lower than negative controls, corroborating once again the little effect of *Cd*Ag in the activation of neutrophils and on their consequent scarce innate immune responses.

For the first time our data reflects the low grade activation of bovine neutrophils in response to the ruminal *C. daubneyi* trematode. We here demonstrate soluble *Cd*Ag and *Cd*-eggs are weak inducers of bovine NETosis and general neutrophil activation. Such outcome could be explained by recent transcription level analyses performed to different stages of *C. daubneyi*, showing distinct patterns of temporal gene expression that correlate with both their development and maturity and niche within the host (9). The results show that *C. daubneyi* secretory proteins are developmentally regulated and correlate with the migration of the fluke within the gastrointestinal tract of the ruminant host (9). For example, in adult fluke secretome, more than 35% of the total proteins analyzed were defense-associated proteins and CdHDM-3 (trematode specific helminth defense molecules clade 3) was found to be the most abundant protein detected. Other proteins enriched in the adult fluke secretome, with potential roles in defense mechanisms, included GST, thioredoxin, and peptidoglycan-recognition protein (9) might be capable to modulate or even neutrophil-derived effector mechanisms.

In conclusion, *C. daubneyi* barely activates bovine neutrophils, which might suggest that the release of *C. daubneyi*-specific molecules (i. e. ES antigens, proteases, or nucleases) could interfere with neutrophil-related effector mechanisms. Further *ex vivo* analyses will clarify if neutrophils are also involved in the pathogenesis of the disease during retrograde mucosal migration by demonstrating neutrophil recruitment into affected duodenal mucosa.

## Data Availability

The datasets presented in this study can be found in online repositories. The names of the repository/repositories and accession number(s) can be found in the article/**Supplementary Material**.

## Supplementary material

Supplementary Table 1 Amplified sequences of IT2 and COX1 and comparison with sequences available at GenBank that confirm that the parasite specimens belong to *Calicophoron daubneyi*.

Supplementary Figure 1 Exposure of bovine neutrophils to *Calicophoron daubneyi* antigen (*Cd*Ag 0.01-100 µg/mL) induce extracellular acidification. **(A)** Extracellular acidification rates (ECAR) show marked increase with 10 and 100 µg/mL, while lower concentrations show no alterations (*n* = 3). **(B)** Oxygen consumption rates (OCR) remained unaltered after injection of *Cd*Ag 10 µg/mL into the wells, after 5 baseline measurements until the end of the experiment (*n* = 3, mean). **(C)** AUC of OCR is not significantly higher in exposed neutrophils, even with strong individual variability (*n* = 3). **(D)** ECAR of *Cd*Ag 10 µg/mL stimulated neutrophils show no significant increase (*n* = 3) also not observed with AUC **(E)**.

Supplementary Figure 2 H_2_O_2_ production was not altered by stimulation with *Cd*Ag. **(A)** *Cd*Ag 10 µg/mL did not alter H_2_O_2_ production, while *Cd*Ag 100 µg/mL induced changes were the same magnitude of effect that was observed when *Cd*Ag 100 µg/mL alone was tested, showing that *Cd*Ag 100 µg/mL is also not capable of inducing any H_2_O_2_ production.
