# Supplementary material for: Cellular immune responses of bovine polymorphonuclear neutrophils to Calicophoron daubneyi
Source: Front Immunol. 2025 Feb 13;16:1515419. doi: 10.3389/fimmu.2025.1515419 (PMC11865088; doi:10.3389/fimmu.2025.1515419)
Supplement: Supplementary Table 1 — Amplified sequences of IT2 and COX1 and comparison with sequences available at GenBank that confirm that the parasite specimens belong to Calicophoron daubneyi. [file DataSheet1.pdf]

Supplementary table 1

**Sequence ID**	**Parasite Gene/Region**	**Sequence**	**Length (bp)**	**Closest Match Description**	**Percent Identity**	**Query Cover**	**GenBank Reference**
Seq 1	ITS2	CTGCTTTGAACATCGACATCTTGAACGCACATTGCGGCCACGGGTTTTCCTGTGGCCACGCCTG<br>TCCGAGGGTCGGCTTATAAACTATCACGACGCCCAAAAAGTCGTGGCTTGGAATCTGCCAGCTG<br>GCGTGATTTCCTCTGTGGTTCGCCACGTGAGGTGCCAGATCTATGGCGTTTTCCTAATGTCTCC<br>GGACACAACCGCGTCTTGCTGGTAGCACAGACGAGGGTGTGGCGGTAGAGTCGTGGCTCAGTTA<br>ACTGTAATGGTAGCACGCTCTGCTGTTGTGCCTTTGAATGGTAACTGGTTTGAGATGCTATTGC<br>TGTCCGTCCAATCATGATCACCTACTGTGGTGTTCTGTTACCTGACCTCGGATCAGACGTGAAT<br>ACCCGCTGAACTTAAGCATATCACTAAGCGGAGGAAAAAGAACCTAACC	433	*Calicophoron daubneyi*	100.00%<br><br>100.00%	96%<br><br>95%	PP957469.1 - Nosal, P. and Kowal, J. (2024)<br><br>KP201674.1 - Chryssafidis, A. L., Fu,Y., De Waal,T. and Mulcahy,G.(2015)
Seq 2	ITS2	ATTCACGGTCTGATCCGAGGTCAGGTAACAGAACACCACAGTAGGTGATCATGATTGGACGGAC<br>AGCAATAGCATCTCAAACCAGTTACCATTCAAAGGCACAACAGCAGAGCGTGCTACCATTACAG<br>TTAACTGAGCCACGACTCTACCGCCACACCCTCGTCTGTGCTACCAGCAAGACGCGGTTGTGTC<br>CGGAGACATTAGGAAAACGCCATAGATCTGGCACCTCACGTGGCGAACCACAGAGGAAATCACG<br>CCAGCTGGCAGATTCCAAGCCACGACTTTTTGGGCGTCGTGATAGTTTATAAGCCGACCCTCGG<br>ACAGGCGTGGCCACAGGAAAACCCGTGGCCGCAATGTGCGTTCAAGATGTCGATGTTCAAAGCA<br>GTATGCAGTTCACATTAATTCACACAGTTGGCTGCGCTCTCAATTCGACACA	436	*Calicophoron daubneyi*	99.77%<br><br>99.76%	98%<br><br>95%	PP957469.1 - Nosal, P. and Kowal, J. (2024)<br><br>KP201674.1 - Chryssafidis, A. L., Fu,Y., De Waal,T. and Mulcahy,G.(2015)
Seq 3	COX1	ACAAACCGTCCCTCAACTTATTAATGAAAAGAATCTCAACAACACCTCTGTTTTACAAAAACAA<br>CATTCTACCCCCTAAACTACCTTTTCTCTCACCCTTAAAACCAACGACCAGAACCACTCATATA<br>TACAACATGCTGAGGCACAGGTAACACAACAACTTTCAAAGAAACATTTGAACTACCCCAAGCA<br>GCCACTACAACATTATGTACAACCAAAGACTCCCACAAAATAAGTACCAAAAAAAAAGCACTAA<br>TCACCGACAAAAAAGCACCAAAAGACGCCAAACTCTCCAACCAAAAAAAATCAGGATTATAAAC<br>ACAAACACGCCGTGGCAAACCACACAAACCAAAATAATGCATAGGAAAAAAACACAAATTAAAC<br>CCCACCATTGAACATAACCAATGACCTTGCAACATATACTTATTCAAACTCAAACCAACTATCA<br>AAGGTCACCACCACACCAATGAAATCACAACAGCACTATATGACCCTAGTGATAACACATAATG<br>AAAATGAGCAACAACAAATCAGGTATCATGCACCATAGAATCTAACACACAAGATGACAATACT<br>ATTCCCGTCACACCACCCATAGTGAACAACACAATAAAACCCATAATCCACCACACAACAGGAT<br>CTCAAACACGTACGCCAGTACCACTCAACATATATAACCAAGAAAAAACCTTTATACCTGTCGG<br>TATACCAATTACCATAGAAACAGAACTAAAAAACACAGATGTCTTAACATCTAACCCTACCATA<br>AACATATGATGTGCTCACACAACACTACCTAAACAAACAATAGAAGCCATAGCAAATACCAAAC<br>CATAATAACCAAACAACGAATCTTGATTTCTCATCCTCATACAAATATGCCTTACAGCCCCAAA<br>CCCCGGCAATATCAGTACATAAACCTCA	924	*Calicophoron daubneyi*	100.00%<br><br>93.30%	99%<br><br>98%	KP979656.1 - Bauer,C., Hirzmann,J. and Koehler,K. (2015)<br><br>MK052977 - Bauer,C., Hirzmann,J. and Koehler,K. (2019)
Seq 4	COX1	TTGGGGCTGTAAGGCATATTTGTATGAGGATGAGAATCAAGATTCGTTGTTTGGTTATTATGGC<br>TTGGTATTTGCTATGGCTTCTATTGTTTGTTTAGGTAGTGTTGTGTGAGCACATCATATGTTTA<br>TGGTAGGGTTAGATGTTAAGACATCTGTGTTTTTTAGTTCTGTTTCTATGGTAATTGGTATACC<br>GACAGGTATAAAGGTTTTTTCTTGGTTATATATGTTGAGTGGTACTGGCGTACGTGTTTGAGAT<br>CCTGTTGTGTGGTGGATTATGGGTTTTATTGTGTTGTTCACTATGGGTGGTGTGACGGGAATAG<br>TATTGTCATCTTGTGTGTTAGATTCTATGGTGCATGATACCTGATTTGTTGTTGCTCATTTTCA<br>TTATGTGTTATCACTAGGGTCATATAGTGCTGTTGTGATTTCATTGGTGTGGTGGTGACCTTTG<br>ATAGTTGGTTTGAGTTTGAATAAGTATATGTTGCAAGGTCATTGGTTATGTTCAATGGTGGGGT<br>TTAATTTGTGTTTTTTTCCTATGCATTATTTTGGTTTGTGTGGTTTGCCACGGCGTGTTTGTGT<br>TTATAATCCTGATTTTTTTTGGTTGGAGAGTTTGGCGTCTTTTGGTGCTTTTTTGTCGGTGATT<br>AGTGCTTTTTTTTTGGTACTTATTTTGTGGGAGTCTTTGGTTGTACATAATGTTGTAGTGGCTG<br>CTTGGGGTAGTTCAAATGTTTCTTTGAAAGTTGTTGTGTTACCTGTGCCTCAGCATGTTGTATA<br>TATGAGTGGTTCTGGTCGTTGGTTTTAAGGGTGAGAGAAAAGGTAGTTTAGGGGGTAGAATGTT<br>GTTTTTGTAAAACAGAGGTGTTGTTGAGATTCTTTTCATTAATAAGTTGAGGGAC	887	*Calicophoron daubneyi*	99.77%<br><br>93.71%	100%<br><br>98%	KP979656.1 - Bauer,C., Hirzmann,J. and Koehler,K. (2015)<br><br>MK052977 - Bauer,C., Hirzmann,J. and Koehler,K. (2019)
