# Supplementary figures and images for: Cellular immune responses of bovine polymorphonuclear neutrophils to Calicophoron daubneyi
Source: Front Immunol. 2025 Feb 13;16:1515419. doi: 10.3389/fimmu.2025.1515419 (PMC11865088; doi:10.3389/fimmu.2025.1515419)

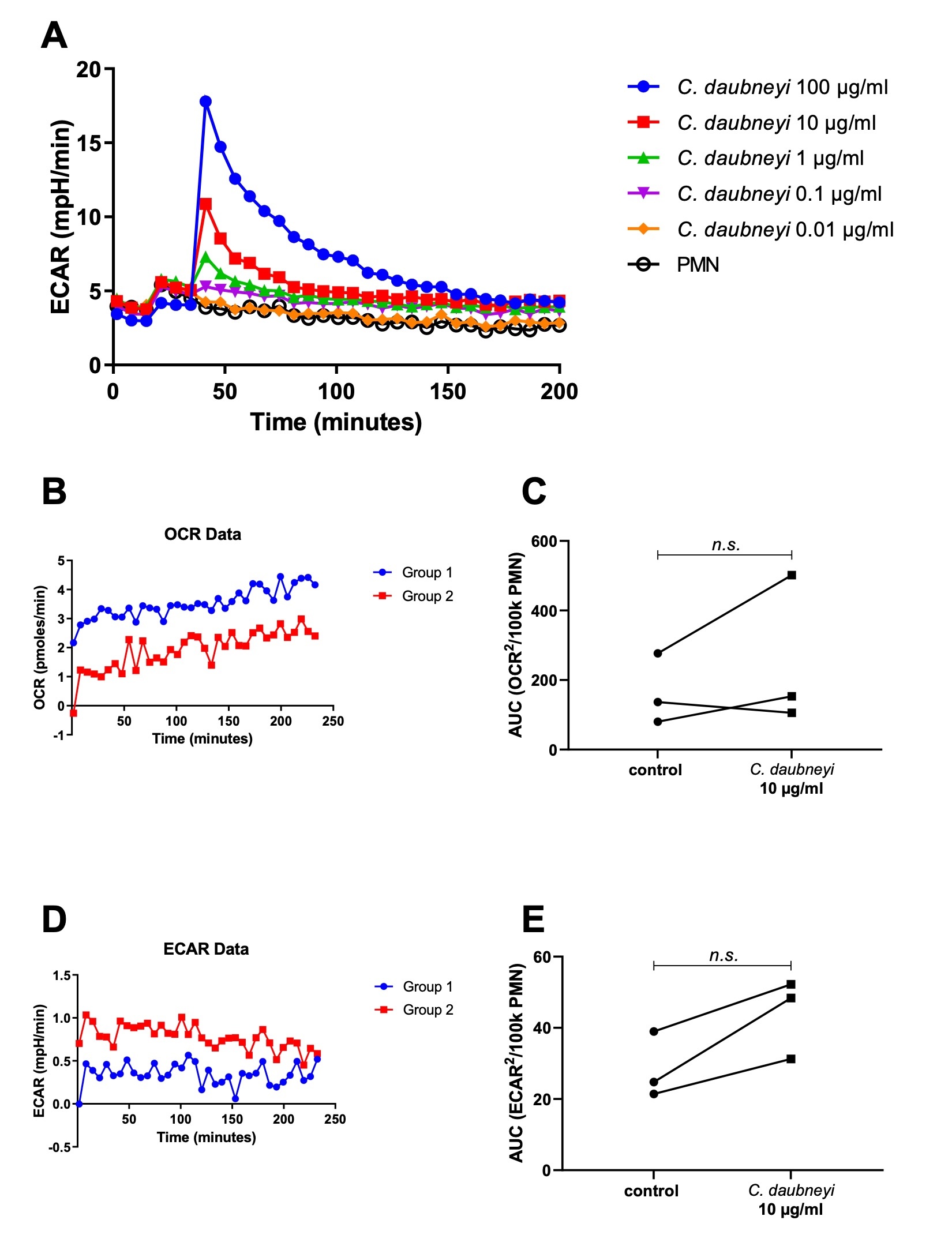

Supplement: Supplementary Figure 1 — Exposure of bovine neutrophils to Calicophoron daubneyi antigen (CdAg 0.01-100 µg/mL) induce extracellular acidification. (A) Extracellular acidification rates (ECAR) show marked increase with 10 and 100 µg/mL, while lower concentrations show no alterations (n = 3). (B) Oxygen consumption rates (OCR) remained unaltered after injection of CdAg 10 µg/mL into the wells, after 5 baseline measurements until the end of the experiment (n = 3, mean). (C) AUC of OCR is not significantly higher in exposed neutrophils, even with strong individual variability (n = 3). (D) ECAR of CdAg 10 µg/mL stimulated neutrophils show no significant increase (n = 3) also not observed with AUC (E). [file Image1.jpeg]

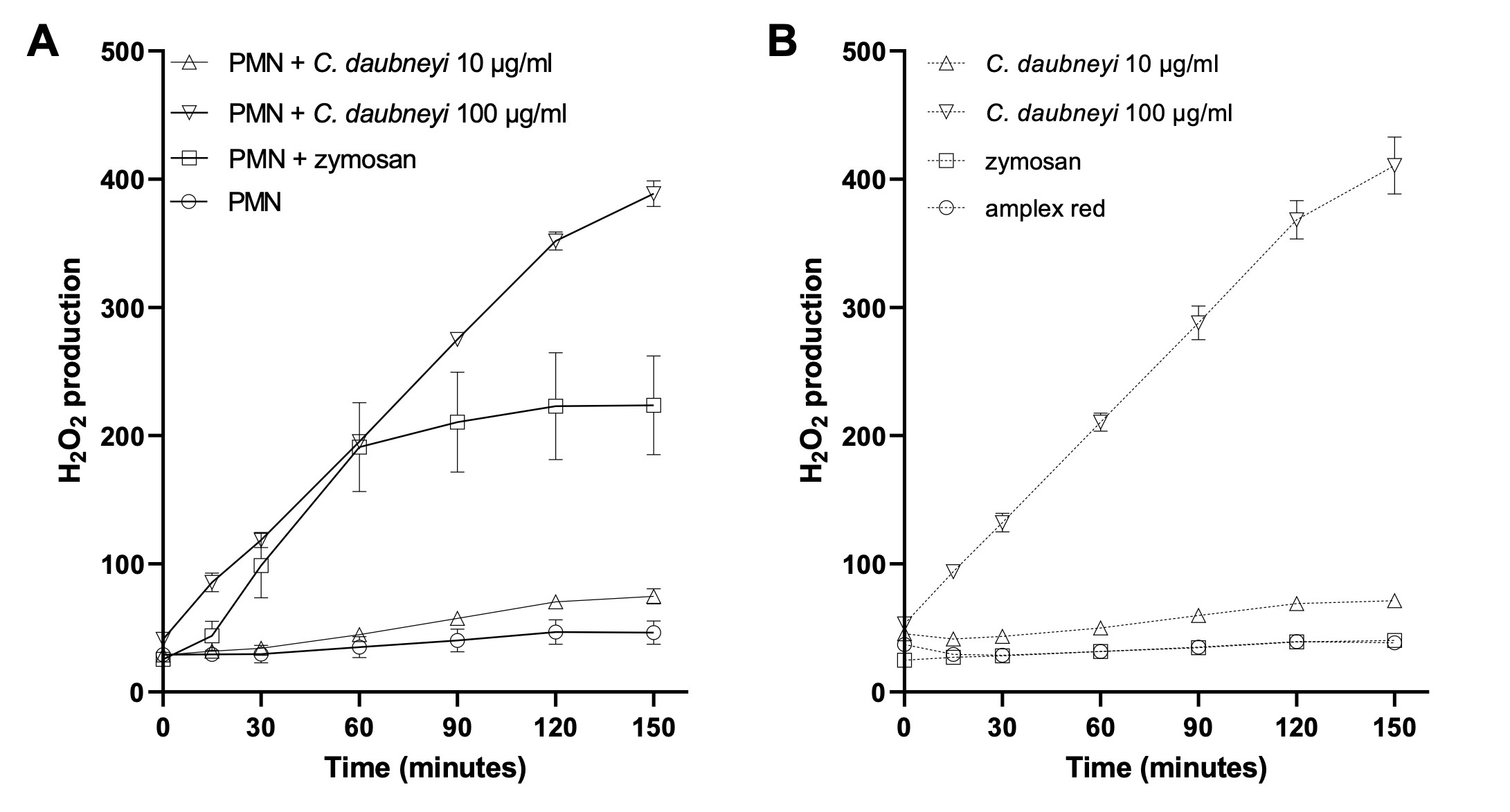

Supplement: Supplementary Figure 2 — H2O2 production was not altered by stimulation with CdAg. (A) CdAg 10 µg/mL did not alter H2O2 production, while CdAg 100 µg/mL induced changes were the same magnitude of effect that was observed when CdAg 100 µg/mL alone was tested, showing that CdAg 100 µg/mL is also not capable of inducing any H2O2 production. [file Image2.jpeg]
